# Hemisection: A Window of Hope For Freezing Tooth

**DOI:** 10.1155/2012/390874

**Published:** 2012-08-14

**Authors:** Usha Radke, Rajesh Kubde, Aditi Paldiwal

**Affiliations:** ^1^Department of Prosthodontics, VSPM's Dental College & Research Centre, Nagpur 440019, India; ^2^Department of Conservative & Endodontics, VSPM's Dental College & Research Centre, Nagpur 440019, India

## Abstract

Advances in dentistry, as well as the increased desire of patients to maintain their dentition, have led to treatment of teeth that once would have been removed. Mandibular first molars are the most commonly extracted teeth due to dental caries and periodontal disease. These teeth are the major standpoint for occlusion, and also have a wide pericemental area. Hence, any defect in the root either mesial or distal, extraction is the most common treatment planned. Under specific conditions, only the diseased part of the tooth can be extracted after an endodontic treatment. A modified fixed partial denture design is fabricated to splint the remaining portion of the tooth to adjacent teeth. This procedure though daunting can be easily achieved and maintained successfully.

## 1. Introduction 

 A terminal abutment molar with extensive decay may be unsuitable for restoration. In such cases, the treatment options are limited and may include a removable partial denture or a dental implant to replace the missing tooth [1]. Alternatively, if the decay is limited to one root, a hemisection procedure may be possible. Periodontal, prosthodontics, and endodontic assessment for appropriate selection of cases is important. From a periodontal perspective, this procedure is indicated if there is severe bone loss limited to one root or involvement of class III furcations that could produce a stable root after hemisection. This procedure is also appropriate if the patient is unable to perform appropriate oral hygiene in the area. Extensive exposure of the roots because of dehiscence is another indication for excision of one root. From a restorative standpoint, treatment by hemisection is indicated for failure of an abutment within a fixed prosthesis, provided a portion of the tooth can be retained to act as the abutment for the prosthesis. Other indications include vertical root fracture confined to a single root of a multirooted tooth or any severe destructive process that is confined to a single root, including caries, external root resorption, and trauma. Contraindications include the presence of a strong abutment tooth adjacent to the proposed hemisection, which could act as an abutment to a prosthesis. The remaining root may be inoperable for the necessary root canal treatment. Also, fusion or proximity of the roots may prevent their separation [2].

 Hemisection refers to sectioning of a mandibular molar into two halves followed by removal of the diseased root and its coronal portion [3]. The retained root is endodontically treated and the furcations area is made self-cleansable by removing the lip of root carefully. Since hemisected teeth fail by root fractures, it is important to restore them adequately by an extracoronal restoration [4]. It is indicated where one of the root of molar is unsalvageable due to caries, periodontitis, or iatrogenic mishaps [5]. It is thus a conservative option with acceptable prognosis [6].

## 2. Case Report 

**Figure 1:**
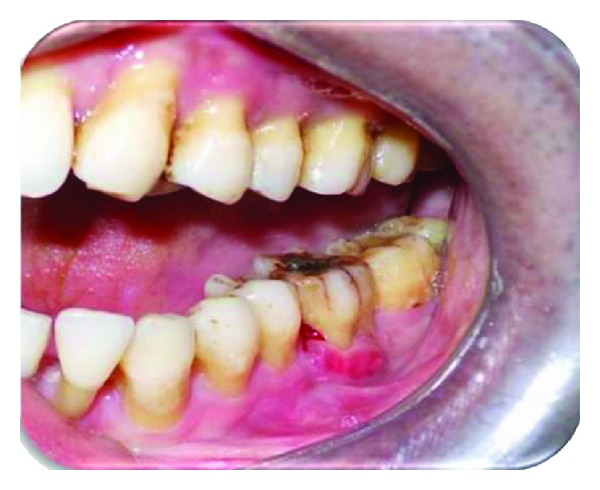
Preoperative view.

 A 35-year-old male patient, reported to the Department of Periodontics, with the chief complaint of loose tooth and pain in lower left back tooth region (Figure 1). Pain was dull aching and intermittent in nature, which aggravated on mastication. On further enquiry, patient did not give any significant medical and previous dental history. Extra oral examination revealed no abnormality. 

 On intraoral examination, it was found that patient had fair oral hygiene. On probing lower left mandibular first molar, a periodontal pocket of 8–10 mm was found on buccal and distal surfaces along with grade III furcation involvement. Also the tooth showed grade II mobility and was sensitive to percussion. IOPA showed grade III furcation defect with periodontal bone loss more along the distal root as compared with mesial root and periapical rarefaction with both the roots. Periodontal support of mesial root of 36 was good. Interproximal bone loss was seen between 36 and 37. Periodontal prognosis with 36 was good and vitality test was positive. Thus, it was diagnosed as chronic generalised gingivitis and localized periodontitis associated with lower left mandibular first molar. Treatment options included extraction of 36 followed by placement of implant, fixed partial denture or removable partial denture. Patient did not wish to have the tooth removed, so a conservative treatment option was opted which included hemisection of the distal root of 36 followed by prosthetic replacement.

## 3. Treatment Procedure 

 Diagnostic impressions were made with irreversible hydrocolloid impression material. 

## 4. Endodontic Phase 

**Figure 2:**
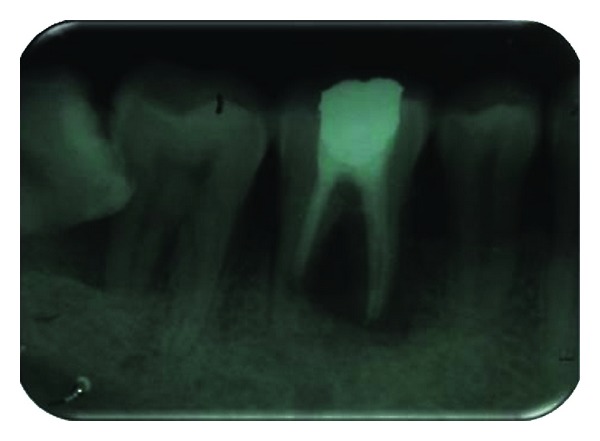
Intentional RCT done.

**Figure 3:**
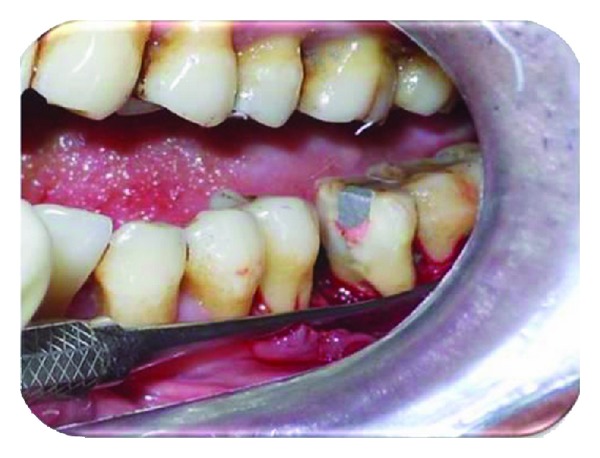
Hemisection of mesial root.

 Endodontic phase involved intentional root canal treatment of 36 in a conventional manner (Figure 2). After 15 days of obturation, hemisection was carried out (Figure 3). 

## 5. Periodontic Phase 

**Figure 4:**
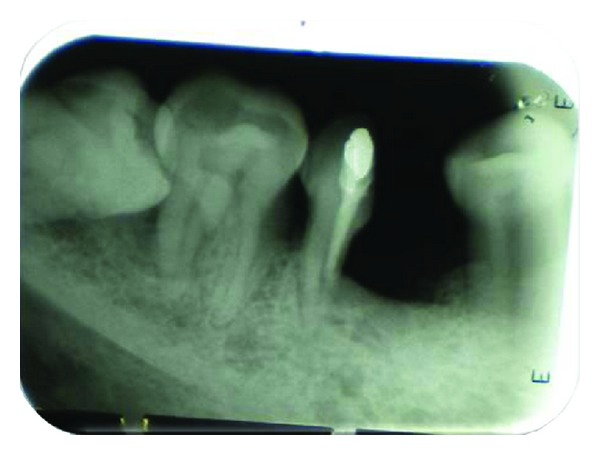
Bone formation after 3 months.

 After appropriate local anaesthesia, a crevicular incision was made from first premolar to second molar region. A full thickness mucoperiosteal flap was elevated to provide adequate access for visualization and instrumentation and minimize surgical trauma. After reflection of flap, bony defect was evident and curettage and debridement were done. A long shank tapered fissure carbide bur was used to make vertical cut facio-lingually towards the bifurcation area and mesial root was extracted. Care was taken not to traumatize bone and adjacent tooth while removing the mesial root. Debridement and irrigation of the socket along with thorough root planning of distal root was performed. Odontoplasty was performed to remove the developmental ridges, and mesial aspect of distal root was contoured in such a way so as to facilitate oral hygiene measures. Socket preservation was done by grafting the extraction site with “Fisiograft.” Then buccal and lingual flaps were approximated to cover the graft. Sutures were placed, and COE pack surgical dressing was done. The surgical site was then allowed to heal with no occlusal stress on distal root for 4 weeks. Patient was recalled after 3 months. IOPA revealed good bone regeneration which indicates good uptake of the graft (Figure 4). Then, the restoration of hemisected tooth was planned with fixed partial denture in relation to 35, mesial root of 36 and 37. 

## 6. Prosthodontic Phase (Restoration ****of Hemisected Tooth) 

**Figure 5:**
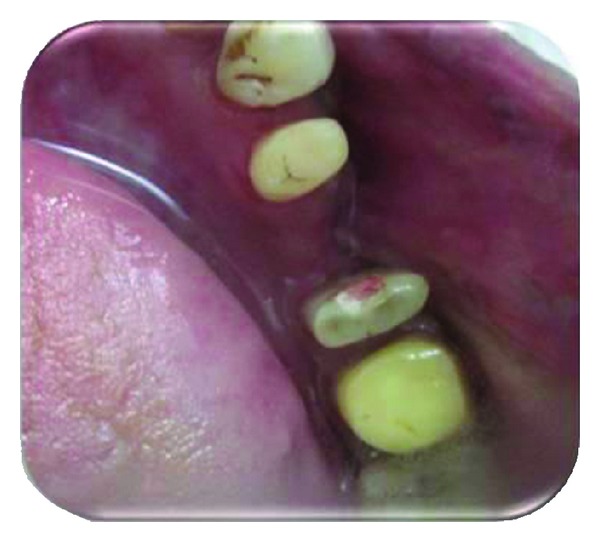
Tooth preparation done.

**Figure 6:**
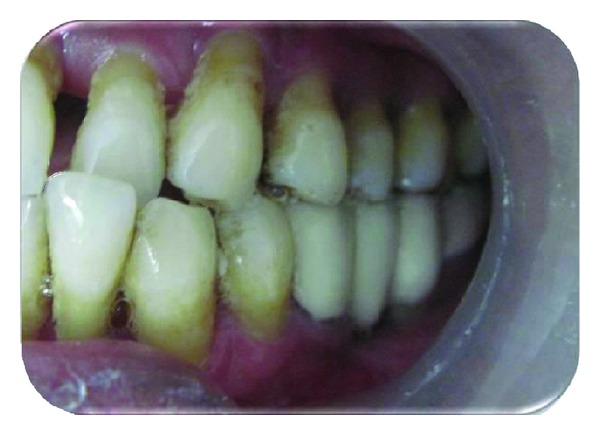
Final prosthesis cemented.

Diagnostic impressions were made with irreversible hydrocolloid impression material and diagnostic casts were obtained. Face bow record was made and transferred to a semi-adjustable articulator and maxillary cast was mounted. Mandibular diagnostic cast was mounted using interocclusal record, to check for any occlusal prematurities and interferences and necessary occlusal corrections were carried out. Tooth preparation was done in relation to 35, distal root of 36 and 37 to receive a porcelain fused to metal restoration (Figure 5). The margin on distal surface of 37 was placed approximately 3-4 mm above the gingival margin as the tooth was mesially tilted or else excessive tooth structure would have been lost in order to create a favourable path of insertion. This will also help in maintenance of gingiva by making it self-cleansable. Final impression was made using putty-reline technique and master cast was obtained. Mandibular master cast was mounted using interocclusal record. Wax pattern was fabricated, sprued, and invested. Casting procedure was carried out using standard techniques. Metal framework was tried in the patient's mouth followed by ceramic build up and bisque try in. Final prosthesis was cemented using glass ionomer cement (Figure 6). Post cementation instructions regarding periodontal maintenance were given. Recall was done periodically to assure the healing and success of the restoration. 

## 7. Discussion 

 Periodontal, prosthodontics, and endodontic assessment for appropriate selection of cases is important. Buhler stated that hemisection should be considered before every molar extraction [7], because it provides a good, absolute, and biological cost saving alternative with good long term success. The treatment options to replace severely damaged and possibly unrestorable teeth include removable partial denture, fixed partial denture, and dental implant. A guiding principle should be to try and maintain what is present [8]. The use of hemisection to retain a compromised tooth offers a prognosis comparable to any other tooth with endodontic treatment. 

### 7.1. Endodontic Phase

 Endodontic treatment was performed first because in case, if the tooth cannot be treated endodontically or if there is an endodontic failure, the case will be contraindicated for hemisection. 

### 7.2. Periodontic Phase

Four critical factors in selecting molar for hemisection are following [9].

1. Root Divergence. Ideally the resected root should have generous root divergence, as close root proximity will make surgery difficult.
2. Root Form. Roots of mandibular molars show concavity, mostly on distal root. Therefore, odontoplasty should be performed to provide a proper contour.
3. Location of Furcation. Closer the furcation opening to the cemento-enamel junction, better the prognosis for retained root.
4. Remaining Root Attachment. is critical to evaluate; as cylindrical, ovoid, and long root serves as an excellent abutment.

*Objectives*

1. To facilitate maintenance.
2. To prevent further attachment loss.
3. To obliterate furcation defects as a periodontal maintenance problem.

### 7.3. Prosthodontic Phase

When the tooth lose part of its root support, it will require a restoration to permit it to function independently or serve as an abutment for fixed partial denture or splint. Thus, restoration is required for function and stabilization of occlusion. 

 Points to consider while fabricating the prosthesis: Restoration can contribute to periodontal destruction, if margins are defective or if nonocclusal surfaces do not have physiologic form. An improperly shaped occlusal contact area converts acceptable forces into destructive forces leading to ultimate failure of hemisection. Hemisected abutment are given a taper greater than 6–10 degree to have a path of insertion compatible with the anterior abutment and to compensate for this buccal and lingual grooves are placed in the abutment. Occlusal table is reduced in size in order to decrease the forces on the retained hemisected root. Cuspal inclines are made less steep to reduce laterally directed forces and eliminate the nonworking contacts. Retained root is restored as premolar which helped to reduce the masticatory load. Stein noted that “esthetic permitting, the sanitary pontic is the best design for posterior region” [10].

 The keys to long term success include thorough diagnosis, selection of patients with good oral hygiene, careful surgical and restorative management. Hemisection may be a suitable alternative to extraction and implant therapy and should be discussed with patients during consideration of treatment options. 

## 8. Conclusion 

 Therapeutic planning, operative sequence, and pluridisciplinarity exerted in this case illustrate the importance of specialized knowledge and professional communication. Hemisection is a baton for the extracting teeth. Careful case selection determines the long term success of the procedure. 

## References

[1] Koka S (2006). Is an implant-supported restoration better than a fixed partial denture to replace single missing teeth?. *Compendium of Continuing Education in Dentistry*.

[2] Weine FS (1996). *Endodontic Therapy*.

[3] Kost WJ, Stakiw JE (1991). Root amputation and hemisection. *Journal of the Canadian Dental Association*.

[4] Rapoport RH, Deep P (2003). Traumatic hemisection and restoration of a maxillary first premolar: a case report. *General Dentistry*.

[5] Saad MN, Moreno J, Crawford C (2009). Hemisection as an alternative treatment for decayed multirooted terminal abutment: a case report. *Journal of the Canadian Dental Association*.

[6] Kurtzman GM, Silverstein LH, Shatz PC (2006). Hemisection as an alternative treatment for vertically fractured mandibular molars. *Compendium of Continuing Education in Dentistry*.

[7] Desanctis M, Murphy KG (2000). The role of resective periodontal surgery in the treatment of furcation defects. *Periodontology 2000*.

[8] Nowakowski AT, Serebnitski A, Pesun IJ (2010). Hemisection as a treatment option: a case report. *Oral Health*.

[9] Parmar G, Vashi P (2003). Hemisection: a case report and review. *Endodontology*.

[10] Schmitt MS, Brown HF (1987). The hemisected mandibular molar. A strategic abutment. *Journal of Prosthetic Dentistry*.

